# Large Language Model–Enabled Editing of Patient Audio Interviews From “This Is My Story” Conversations: Comparative Study

**DOI:** 10.2196/80205

**Published:** 2026-01-09

**Authors:** Bikram Bains, Sampath Rapuri, Edgar Robitaille, Jonathan Wang, Arnav Khera, Catalina Gomez, Eduardo Reyes, Cole Perry, Jason Wilson, Elizabeth Tracey

**Affiliations:** 1 Department of Biomedical Engineering, Johns Hopkins University, Baltimore, MD, United States; 2 School of Medicine, Johns Hopkins University, Baltimore, MD, United States; 3 Department of Computer Science, Johns Hopkins University, Baltimore, MD, United States; 4 Technology Innovation Center, Johns Hopkins Medicine, Baltimore, MD, United States; 5 Division of Spiritual Care and Chaplaincy, Johns Hopkins Medicine, 1800 Orleans St, Baltimore, MD, 21218, United States, 1 410 215 7749

**Keywords:** audio recording, communication, This Is My Story, TIMS, distress reduction, empathy, patient interview, provider/patient communication, large language model

## Abstract

**Background:**

This Is My Story (TIMS) was started by Chaplain Elizabeth Tracey to promote a humanistic approach to medicine. Patients in the TIMS program are the subject of a guided conversation in which a chaplain interviews either the patient or their loved one. They are asked four questions to elicit clinically actionable information that has been shown to improve communication between patients and medical providers, strengthening medical providers’ empathy. The original recorded conversation is edited into a condensed audio file approximately 1 minute and 15 seconds in length and placed in the electronic health record where it is easily accessible by all providers caring for the patient.

**Objective:**

TIMS is active at the Johns Hopkins Hospital and has shown value in assisting with provider empathy and communication. It is unique in using audio recordings to accomplish this purpose. As the program expands, there exists a barrier to adoption due to limited time and resources needed to manually edit audio conversations. To address this, we propose an automated solution using a large language model to create meaningful and concise audio summaries.

**Methods:**

We analyzed 24 TIMS audio interviews and created three edited versions of each: (1) expert-edited, (2) artificial intelligence (AI)–edited using a fully automated large language model pipeline, and (3) novice-edited by two medical students trained by the expert. A second expert, blinded to the editor, rated the audio interviews in a randomized order. This expert scored both the audio quality and content quality of each interview on 5-point Likert scales. We quantified transcript similarity to the expert-edited reference using lexical and semantic similarity metrics and identified omitted content relative to that same expert interview.

**Results:**

Audio quality (flow, pacing, clarity) and content quality (coherence, relevance, nuance) were each rated on 5-point Likert scales. Expert-edited interviews received the highest mean ratings for both audio quality (4.84) and content quality (4.83). Novice-edited scored moderately (3.84 audio, 3.63 content), while AI-edited scored slightly lower (3.49 audio, 3.20 content). Novice and AI edits were rated significantly lower than the expert edits (*P*<.001), but not significantly different from each other. AI and novice-edited interview transcripts had comparable overlap with the expert reference transcript, while qualitative review found frequent omissions of patient identity, actionable insights, and overall context in both the AI and novice-edited interviews. AI editing was fully automated and significantly reduced the editing time compared to both human editors.

**Conclusions:**

An AI-based editing pipeline can generate TIMS audio summaries with comparable content and audio quality to novice human editors with one hour of training. AI significantly reduces editing time and removes the need for manual training; with further validation, it could offer a solution to scale TIMS to a large range of health care settings.

## Introduction

Recent statistics show that health worker burnout is a widespread issue [1]. A 2022 Centers for Disease Control and Prevention report found that 46% of health workers “often” or “very often” felt burned out, increasing from 32% in 2018. Physicians in the United States also report similarly high burnout rates (56% in 2021, 53% in 2022, and 48% in 2023), with an all-time high physician burnout rate of 63% during the pandemic [2,3]. Some reasons for this burnout include excessive work hours, administrative burdens (such as electronic health record documentation), insufficient support staff, and limited organizational and leadership support [4-8]. These chronic stresses impact both patients and clinicians. For example, Andhavarapu et al [9] mentioned that symptoms of depression, anxiety, and posttraumatic stress disorder were reported in 34% of the health care workers surveyed (while 14% reported severe posttraumatic stress disorder), with the highest prevalence among nursing staff (42.8%) and physicians (25.2%). Similarly, the National Academies’ 2019 report found that between 35% and 54% of US nurses and physicians and 45% to 60% of medical students and residents experience substantial burnout symptoms throughout their careers [10].

Empathy can serve as a solution, reducing widespread symptoms of burnout while promoting professional fulfillment and strengthening connection with patients [11-14]. Already, health care organizations have recognized the value of empathy and designed personal and patient-centered interventions within their clinical workflows [15]. For example, the This Is My Story (TIMS) program was developed by Chaplain Elizabeth Tracey at the Johns Hopkins Hospital to bring a more patient-centered and empathetic approach to medicine [16]. Patients who participate in the TIMS program take part in a conversation with a chaplain; if the patient is noncommunicative, a chaplain has a conversation with the patient’s loved ones. These conversations are guided by four questions: How do you prefer to be addressed? What brings you joy? What does your medical team need to know to care for you best? What brings you peace?

In the words of Dr Charles Cumming, Director Emeritus of Otolaryngology at Johns Hopkins, *“TIMS is about helping us get back to the proper essence of medicine...it’s essential to get to know the patient if we’re going to be able to help that patient as best we can”* [17]. TIMS conversations have demonstrated clear benefits for clinical communication and empathy, providing an opportunity for meaningful connection with patients to directly target the emotional aspects of burnout [16,18-20]. Past studies by Tracey et al [21] support the positive outcomes the program has had on patients, their families, and the care team. For example, one previous study reported a 74% increase in staff empathy for patients and a 99% improvement in interactions by patients’ loved ones with the medical team. Although it has also been shown to be useful in improving staff empathy and reducing distress by 69%, the process of recording and editing conversations can be labor-intensive [21]. By automating the conversation summarization process, these benefits can be made accessible to a wider range of patients and medical institutions.

In this study, we propose an automated editing pipeline for TIMS interviews using a large language model (LLM) and evaluate whether artificial intelligence (AI)–edited interviews are a viable alternative to manual editing. Because medical students were frequently trained to edit TIMS interviews during the pandemic, they are a reasonable baseline for comparing performance. We designed our analysis around two key hypotheses: (1) that AI-edited interviews maintain similar quality to expert-edited interviews in both audio and content metrics, and (2) that AI-edited interviews can be produced more quickly than interviews produced by expert or novice editors.

## Methods

### Study Design

We used a within‐subjects, single‐group design in which our reviewer evaluated interviews across three independent editing conditions (expert, AI, novice). Editors were eligible if they had professional experience interpreting patient–clinician audio interviews. Two chaplains from the Johns Hopkins Hospital took part in the study. The novice editors were two medical students who joined the study team from the Johns Hopkins School of Medicine, each having completed an hour-long training session on audio‐editing with an expert editor (Chaplain Elizabeth Tracey). The two novice editors edited 12 randomly assigned audio interviews, mirroring the normal workflow for the TIMS initiative without the AI tool.

### Patient Audio Dataset

We used a retrospective dataset of audio recordings from 24 patients admitted to the Johns Hopkins Neurosciences Critical Care Unit, a tertiary intensive care unit for patients with diseases of the central or peripheral nervous systems, admitted from departments such as neurosurgery, neurology, and others.

### AI Editing Pipeline

**Figure 1.**
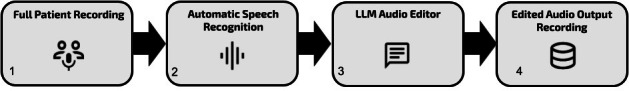
Overview of artificial intelligence–automated workflow for summarizing This Is My Story audio interviews. LLM: large language model.

Audio recordings were first transcribed using Nvidia’s Parakeet-TDT 0.6B v2 automatic speech recognition (ASR) model (Figure 1) [22-24]. ChatGPT-4o [25] processed the transcript using a custom prompt. This prompt asked the model to extract only patient statements that provided essential information about their condition, experiences, feelings, or personal identity. It was also directed to exclude any filler or repetitive content and keep humorous or insightful remarks to preserve patient identity and humanity.

The model was instructed to include the interviewer’s four core questions for the TIMS program while excluding interjections or examples provided by the interviewer. Instructions were given to return a processed transcript that the model estimated would take 1.5 minutes during a TIMS interview. The full model prompt is available in Multimedia Appendix 1. The relevant timestamps identified were then used to splice together the final audio file. Examples of cases in which ASR output might have impacted the LLM output are presented in Table S2 in Multimedia Appendix 2. This process was entirely automated and was run on an NVIDIA GeForce RTX 4090 with 24 GB of RAM.

### Survey Design

To evaluate both audio quality and content quality for each edited TIMS interview, we created an online questionnaire for our reviewer to complete using a 5-point Likert scale (1=poor, 5=excellent) for each question. The survey included questions grouped into audio (natural flow, pausing/spacing, transitions, pacing, overall listenability of the interview) and content (conversation flow, speaker/topic tracking, patient representation, understanding of patient characteristics, preparation for interaction for patient providers/care team, nuance of the patient’s life, relevance of details) quality domains with all details shown in Textbox 1 below. This same survey was used independently for each edited interview for consistent comparison.

Textbox 1. **Summary of domain and survey questions following survey administration and data collection.**

**Audio quality**

1. How natural is the conversation flow?

2. How effective are the pauses and spaces between answers?

3. How smooth are the transitions between questions and answers?

4. How does the pacing of the audio feel overall?

5. Overall, how easy is the interview to listen to?

6. Can you understand the flow of the conversation?

**Content quality**

7. Can you keep track of the speaker and the topic?

8. Is the patient well represented in this conversation?

9. Do you understand the patient’s likes/dislikes (proclivities, idiosyncrasies, etc)?

10. Do you feel prepared to interact with the patient in a meaningful way?

11. How well does the conversation capture the depth and nuance of the patient’s experience?

12. Does the conversation convey the patient’s emotions effectively?

13. How relevant are the details shared during the conversation for understanding the patient’s story?

14. How engaging is the conversation in terms of sustaining your interest in the patient’s story?

The survey was created and administered using Google Forms. Content experts (ET, JW, CG) provided qualitative feedback on an initial draft of the questions. This feedback focused on improving the clarity and relevance of each item. The questions were then refined based on this input to better capture the intended domains of audio and content quality. It is important to note that because the survey instrument is novel, its reliability and validity have not yet been formally established. The survey asked questions 1, 5, 10, and 11 to gauge general sentiment for the conversation being rated, and more pointed questions to understand where the audios may differ in terms of score (Textbox 1). Since assessing the flow of conversation relies on both audio and content quality, a question about it was used for both parts of the survey. The individual survey responses are available in Figure S1 in Multimedia Appendix 3.

Before rating each edited version, the reviewer listened to the corresponding raw interview as a baseline reference to better understand the context of the interview. Before rating each interview, the reviewer was asked to first listen to two calibration audios—one edited poorly that should score low across all questions, and an expert-edited interview that would score highly. This allowed us to set audio quality expectations for each extreme on the survey. The survey was completed independently for each condition, with the order of the audio files randomized by condition for each patient to reduce potential order effects.

Editing durations were recorded automatically for the AI pipeline and self‐reported by each novice editor. Expert editing times were not collected due to the limitations of the retrospective dataset.

### Content Analysis

For each edited interview, we generated text transcriptions to study the differences in content among the three types of edited interviews. Text transcriptions were created using the Parakeet transcription model. The novice- and AI-edited interviews were compared to the expert edits, and three members of the study team analyzed differences. Types of errors were identified for both AI- and novice-edited conditions across all samples. The most common types of errors were then formalized and described in the results (Table 1). Content similarity between each condition and expert edits was quantitatively measured using ROUGE-L, ROUGE-1, ROUGE-2, bidirectional encoder representations from transformers (BERT), and METEOR on interview transcripts following studies of medical document summarization [26,27]. All metrics measure the similarity of words between summaries and assign a score from 0 to 1, with the ROUGE scores measuring lexical overlap and other scores [28]. METEOR and the BERT scores were used to assess the semantic overlap. Both factor in semantic similarity between words rather than the exact word choice. METEOR also assigns a penalty for differences in phrasing.

**Table 1.** Common omissions and inaccurate portrayals by artificial intelligence and novice editors.

Type of error	Artificial intelligence errors	Novice errors
Omission of actionable patient insights	Failure to include specific interests or hobbies of the patient [talking about favorite musical artists bringing her joy]: “She likes Anita Baker, Regina Belle, and Gladys Knight.”	Failure to include information about the patient’s comfort [informing about her medical condition to better care for the patient]: “She has had eczema since she was about three or four, so her skin has to stay moisturized.”
Omission of patient identity and empathy	Failure to include details relevant to understanding the patient’s background [explaining his occupation and hobbies] : “On the church side, he loves to teach. He is a pastor.”	Failure to mention important characteristics about the patient [claiming that her time at Hopkins has made her more independent and resilient]: “She [patient] worked at Johns Hopkins for over 30 years.”
Omission of emotional background	Failure to include framing details relevant for a patient’s background [talking about what brings the patient joy]: “Me [patient’s husband] … We’ve been married 20 years.”	Failure to include details relevant for a patient’s emotional state and anxiety [explaining how he mainly only trusts his partner for everything]: “[He has] a little bit of a trust issue with the medical field.”
Poor narrative fluency	Prompting questions fail to be edited out of the interview: “Introduce yourself and tell me how you’re related to the patient.”	Filler words before prompting questions fail to be edited out of the interview: “That’s great! So, what brings the patient peace?”

Relationships between audio length and content quality were also explored through simple linear regression of each ROUGE metric on the duration of the original interview (in minutes).

### Statistical Analysis

We conducted a Friedman test to compare audio‐quality and content‐quality ratings across conditions, with Bonferroni‐corrected Wilcoxon signed-rank post hoc tests to adjust for multiple comparisons. Editing times were analyzed with an independent‐samples *t* test to test significant differences between the two novice editors. We also examined the relationship between the raw interview length and lexical and semantic score overlap for each editor type using Pearson correlation, testing if each slope differed from zero. We then performed an analysis of covariance with transcript length, editor type, and their interaction term to determine if the slope of the length-overlap relationship differed between AI and novice editors.

### Ethical Considerations

Ethical approval was not required for this study as it involved a secondary analysis of anonymized data. The original data collection was conducted under Johns Hopkins institutional review board review and approval of the studies with informed consent obtained from all subjects; the consent allowed for future data use and any participants who declined this future use were not included in this secondary analysis. This study was conducted in accordance with all local, institutional, national, and international regulations on human subject research.

## Results

### Quantitative Comparison of Editing Quality

**Figure 2.**
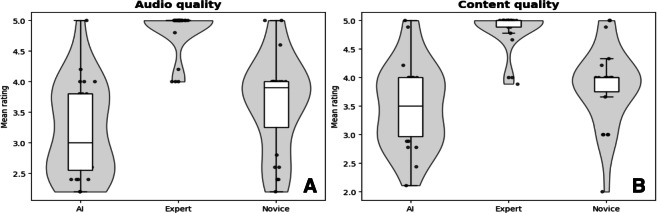
(**A**) A comparison of mean audio quality ratings across the three types of editors aggregated across all 24 audio interviews. (**B**) All editors follow similar trends for the content quality ratings. AI: artificial intelligence.

Across the three types of editors, the mean audio quality ratings were 3.49 (AI-edited), 3.84 (novice-edited), and 4.84 (expert-edited). Similarly, the mean content quality ratings were 3.20 (AI-edited), 3.63 (novice-edited), and 4.83 (expert-edited). The AI-edited interviews demonstrated a higher variability in the rated content quality compared to the novice-edited interviews (SD 0.73 and SD 0.68, respectively). Both interview types were similarly varied in their audio quality ratings (SD 0.77 and 0.78, respectively). Figure 2 highlights the distributions of content and audio quality ratings across each type of edited interview.

We observed significant differences in audio and content quality between the novice- and expert-edited interviews (*P*<.001) as well as between the AI- and expert-edited interviews (*P*<.001). No significant differences were noted between the AI- and novice-edited interviews for either content quality (*P*=.31) or audio quality (*P*=.33). A detailed breakdown of the ratings for each individual survey question across all patient interviews can be found in Figure S1 in Multimedia Appendix 3.

**Figure 3.**
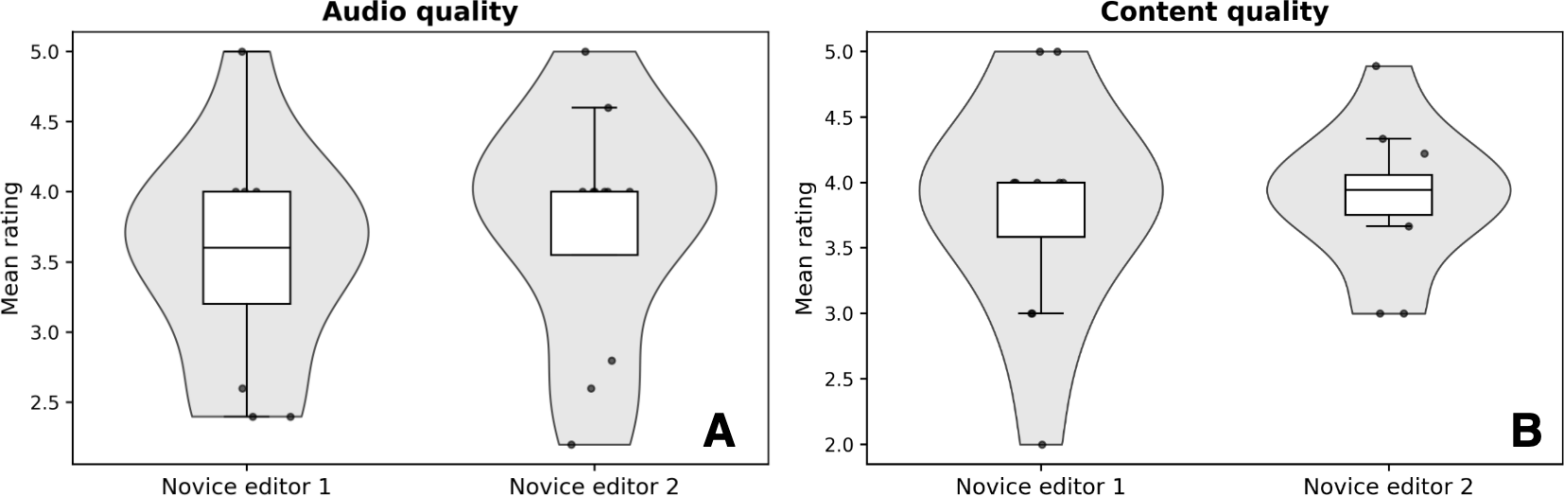
(**A**) A comparison of mean audio quality ratings between the two novice editors, each of whom edited 12 randomly assigned audio interviews. (**B**) Both editors achieved comparable content quality ratings, but the second novice editor exhibited significantly lower variability. All statistical scores of content similarity highlighted the similarities between the artificial intelligence and novice editors, and we report no statistically significant differences between any metric across both types of editors (*P*>.05). Detailed scores across each metric are contained in Table S1 in Multimedia Appendix 2.

**Figure 4.**
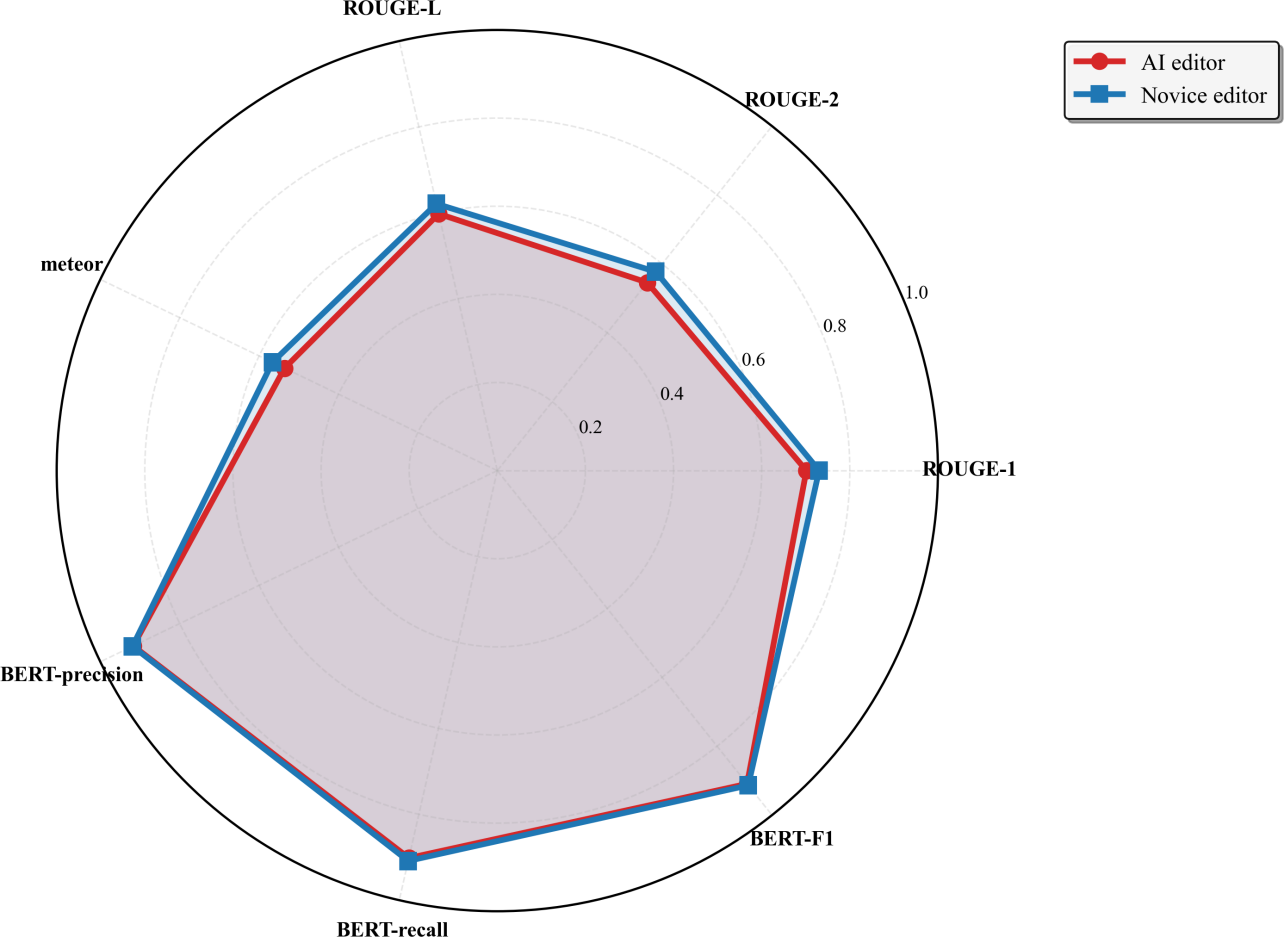
Mean statistical scores across all 24 audio interviews for both the artificial intelligence and novice editors. AI: artificial intelligence. BERT: bidirectional encoder representations from transformers.

To understand the variability between the novice editors, we broke down the differences in rated audio and content quality in Figure 3. Between the two novice editors, we found that novice editor 1 demonstrated a mean content quality score of 3.81 (SD 0.83) and a mean audio quality score of 3.52 (SD 0.76). The second editor’s mean content quality score was measured to be 3.88 (SD 0.52), with a mean audio quality score of 3.75 (SD 0.81). However, neither intragroup difference was significant for both content quality (*P*>.99) and audio quality (*P*=.51). Figure 4 shows the mean statistical scores across all audio interviews for both the AI and novice editors.

### Qualitative Error Analysis

We qualitatively compared the AI- and novice-edited interviews to the expert-edited interviews and found five types of errors repeated across many of the interviews, which are described in Table 1. Many of the errors were similar across the AI- and novice-edited interviews.

### Editing Efficiency

**Figure 5.**
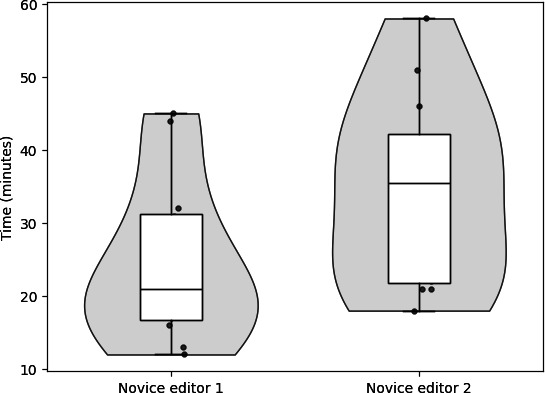
Time to edit each interview between each novice editor.

On average, each novice editor took 29.54 minutes to edit each interview (SD 12.69 min). However, we report a difference (*P*=.06) in the time each novice editor takes, with the first editor taking 24.50 minutes (SD 11.18 min) and the second taking 34.58 minutes (SD 13.08 min) to edit each interview. Figure 5 highlights the variability in the time to edit interviews between novice editors. Mean editing times for the expert editor are unavailable as they were not recorded. Based on anecdotal evidence from the expert editor, each audio interview required around 5 to 10 minutes to edit. In contrast to both the expert and novice editors, our automated AI-editing pipeline took less than 10 seconds from ingestion of the raw audio interview to the saving of the edited interview.

### Impact of Interview Length on Editing Quality

**Figure 6.**
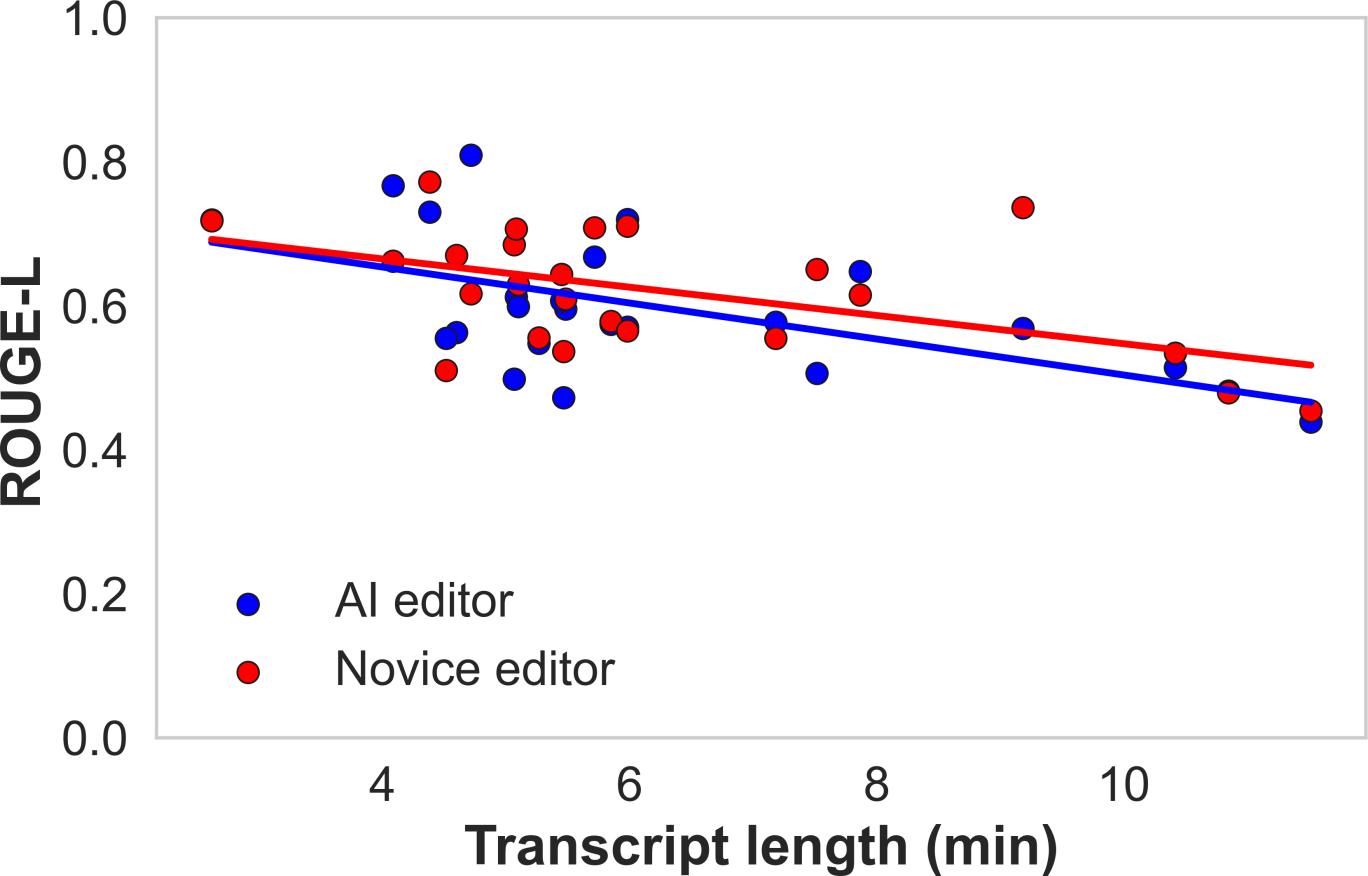
Linear regression of the raw transcript length versus ROUGE-L, reflecting the change in lexical similarity to the expert reference with longer interview times.

We investigated if longer raw audio interviews were associated with changes in the lexical and semantic overlap with the expert reference, as measured by ROUGE-L. For AI-edited interviews, there was a significant negative correlation between transcript length and ROUGE-L scores (*r*=−0.58, *R*^2^=0.34; Figure 6). This same trend was seen for novice-edited interviews, with a negative correlation (*r*=−0.52, *R*^2^=0.27). Both slopes were significantly different from zero (*P*<.05), suggesting longer interviews were associated with lower transcript overlap to expert editor reference for both conditions. Regression plots for ROUGE-1, ROUGE-2, METEOR, and BERT scores showed comparable patterns and are provided in Figure S2 in Multimedia Appendix 3.

## Discussion

### Principal Results

Our study compares the listening experience of patient conversations summarized by an expert editor, a novice editor, and ChatGPT-4o. There was no significant difference in content and audio quality between the AI and novice editors, and both showed varying performance across the samples. Further analysis of the edited transcripts revealed that both of these groups omitted key details. The expert editor had a significantly higher audio and content quality rating than both experimental groups and less variability across samples. Exclusion of natural pauses between phrases and auditory cues to break up conversation led to lower audio quality scores.

### Comparison to Prior Work

As burnout remains high among health care workers, TIMS provides an opportunity for meaningful connections with patients to target the emotional aspect of burnout. However, the manpower needed to edit audio is a limiting factor for this program’s widespread adoption. Although the audio-editing pipeline introduced was originally developed to expand the TIMS program, these results are also broadly relevant to the use of AI in clinical practice and examine a previously unexplored application of LLMs, specifically editing audio content for a medical context. Previous studies have investigated ChatGPT as a clinical decision-making tool, for taking notes, and analyzing literature [26,28-35]. There have also been studies that demonstrate ChatGPT’s capability to elicit empathetic feelings in an emergency setting can even exceed that of clinicians [36-38]. However, the combination of a summarization task to build empathy in a third-party listener has not been investigated. In this study, we aim to evaluate whether an LLM is able to extract emotionally relevant details from a conversation. Additionally, we provide a replicable framework for integrating LLMs in the analysis of patient audio for a broad variety of applications within health care settings. We found that our pipeline faced similar issues raised in previous studies examining ChatGPT’s ability to summarize in a medical context. Kernberg et al [39] reported that 58% of structured medical notes from patient-physician interactions omitted important information. A manual analysis of the transcripts revealed that details considered important to a patient’s story were also often omitted, highlighting a key disadvantage of LLMs in the literature. ChatGPT also tends to vary widely in the quality of responses across the samples. Although this was a shortcoming observed in the novice editors’ performance on audio and content quality (SD 0.68 and SD 0.78), the expert editor’s performance was consistent.

In addition to the survey, ROUGE scores (ROUGE-1, ROUGE-2, ROUGE-L) for the novice and AI editors indicated a high level of word overlap with the expert-edited transcript, with no significant differences between the two groups. High (~0.9) BERT scores (BERT-recall, BERT-precision, BERT-*F*_1_) were also reported, indicating a high degree of semantic overlap that was not necessarily reflected in the ROUGE score. As the length of the audios increased, there was a statistically significant negative correlation between interview length and the ROUGE-L score for both AI and novice editors (*P<*.05), indicating longer interviews tended to have less lexical overlap with the expert reference. This implies that there might be a length of audio that may not be as suitable for AI editing that will become more apparent as longer audios are recorded. These results from an established tool align with the insights from the survey, suggesting some level of construct validity for the survey questions.

### Limitations

There are key limitations beyond the strengths of this study. First, there is no standardized or validated survey instrument available, so the introduction of a novel survey to assess the impact of each audio on a listener was necessary. However, abstract questions concerning “patient representation” or “nuance” are susceptible to subjective interpretation, a weakness that is amplified by our use of a single blinded reviewer. We attempted to standardize these ratings with calibration audios that were developed, but we cannot exclude the possibility that these subjective quality scores were influenced by rater bias. Despite this concern, the consistently high scores awarded to the expert-edited interviews provide some evidence of the survey’s validity, as the rater reliably scored the gold-standard interviews. This survey could be adopted in the future by studies to measure the efficacy of interventions to increase empathy in medicine.

Second, the sample size of the study was also relatively limited, with only 24 samples and 1 recruited rater who was surveyed, which makes the results prone to bias. To build on this work, a larger sample size of patient audio interviews and experienced interview raters should be recruited. Previous volunteers of the program were able to receive iterative feedback on their work over long periods, but the novice editors had approximately 1 hour of training in comparison, so their skills were not as developed. Lastly, we were unable to obtain granular editing time measurements from the expert editor as these were retrospectively edited. However, the AI pipeline’s completion time of under 10 seconds represents a multiple-orders-of-magnitude improvement in efficiency against any manual editing process.

### Future Directions

We have presented the groundwork for an audio transcription and editing pipeline for humanistic patient conversations. Future work should test newer models as they improve, and others that are currently available besides ChatGPT-4o, with the same pipeline. Other strategies to improve performance include fine-tuning the LLM model, using AI agents to summarize the transcript, testing other ASR models, introducing patient-specific contextual metadata, and further prompt engineering to optimize the output. Error propagation was not formally tracked through the entire editing pipeline, but we have hypothesized an association between ASR errors and the final output quality. Future work should investigate these errors.

### Conclusions

We conclude that ChatGPT-4o can create summarized audio files with similar audio and content quality to a novice editor in just a fraction of the time. However, the expert editor outperforms the AI editing pipeline and the novice editors on all metrics. After further validation, this tool could be implemented in the TIMS program to reduce workload and overcome adoption barriers.

## Supplementary material

10.2196/80205 Multimedia Appendix 1 GPT-4o summarization prompt.

10.2196/80205 Multimedia Appendix 2 Supplementary tables.

10.2196/80205 Multimedia Appendix 3 Supplementary figure.

## Abbreviations

AI: artificial intelligence
ASR: automatic speech recognition
BERT: bidirectional encoder representations from transformers
LLM: large language model
TIMS: This Is My Story
