# Supplementary material for: Large Language Model–Enabled Editing of Patient Audio Interviews From “This Is My Story” Conversations: Comparative Study
Source: JMIR Med Inform. 2026 Jan 9;14:e80205. doi: 10.2196/80205 (PMC12788710; doi:10.2196/80205)
Supplement: Multimedia Appendix 1 [file medinform-v14-e80205-s001.docx]

eAppendix 1. GPT-4o Summarization Prompt

Assume you are analyzing a transcript of a doctor-patient conversation.

Extract only the patient's statements that provide essential information about their condition, experiences, or feelings.

- Ignore filler answers and conversation, irrelevant content, repetitive content, or any text not present in the original transcript.
- Introduce the speaker and who is talking as well as their name.
- Extract only the statements that provide essential information about the patient's condition, experiences, or feelings.
- If there is humor in the patient's answer, include it.
- Keep short sequences that may be funny and insightful to give more humanity.
- Extract a combined duration of approximately one minute and thirty seconds of spoken text, taking note to reduce longer answers if the expected spoken time is longer.

Include the questions asked by the interviewer:

1. How does the patient want to be addressed?
2. What brings the patient joy?
3. What does the medical team really need to know about this person in order to give the best possible care?
4. What sustains the patient in times of trouble?

If there is an example of a question answer asked by the interviewer, do not include the example. A common example to NOT include in the final transcript is Question 3, which the interviewer often gives the example that the patient likes their cranberry juice or they get cold easily. This is an example for the interviewee and is not relevant to the final audio, but I still need you to INCLUDE the interviewer's base question in the final transcript.

Do not include interjections from the interviewer as well such as the interviewer laughing or offering other comments to the patient. Still include the interviewer's base question.

Your task is to extract portions of the transcript that are the most useful in understanding the patient as an individual.

Using the timestamps, make sure the combined length of the extracted sentences is around 1 minute and 30 seconds.

THE OUTPUT MUST BE AS FOLLOWS

Output the transcript structure in this JSON format:

{"start": sentence start time, "end": sentence end time, "text": "sentence"}

DO NOT add any extra spaces or filler text and do not include "json" in the output.
