# Supplementary material for: Large Language Model–Enabled Editing of Patient Audio Interviews From “This Is My Story” Conversations: Comparative Study
Source: JMIR Med Inform. 2026 Jan 9;14:e80205. doi: 10.2196/80205 (PMC12788710; doi:10.2196/80205)
Supplement: Multimedia Appendix 2 [file medinform-v14-e80205-s002.doc]

**Table S1.** All statistical scores comparisons between AI and Novice editors.

| Metric | AI Editor Mean | Novice Editor Mean | *P*-value |
| --- | --- | --- | --- |
| ROUGE-1 | 0.701 | 0.730 | 0.551 |
| ROUGE-2 | 0.546 | 0.577 | 0.587 |
| ROUGE-L | 0.598 | 0.621 | 0.798 |
| METEOR | 0.536 | 0.566 | 1.000 |
| BERT-Precision | 0.917 | 0.919 | 1.000 |
| BERT-Recall | 0.902 | 0.908 | 1.000 |
| BERT-F1 | 0.909 | 0.914 | 0.798 |

**Table S2.** Representative ASR transcription errors and hypothesized effects on LLM interpretation

| **Ground Truth** | **ASR Output** | **GPT Output** | **Hypothesized Impacton Final Transcript** |
| --- | --- | --- | --- |
| What kind of music? Oh, he loves all kinds. He’sall over the place. Sometimes he, sometimes it could be Justin Timberlake, or NSYNC, could be the Backstreet Boys, it could be from Usher, you know, R&B. | What kind of music?  Oh, he loves all kinds.  He's all over the place. Sometimes he, sometimes it could be Justin Temblick or NSANC, could be the Bach Street Boys.  It could be from Usher, you know, RB. | {"start":START_TIME,"end":END_TIME,"text":" He loves cartoonsand he loves music "}  [NEGLECTED FAVORITE MUSICAL ARTISTS]  {"start":START_TIME,"end":END_TIME,"text":" So how do you tell what's the best thing to do for him? "} | The misspelling of Justin Timberlake and NSYNC as “Justin Temblick” and “NSANC” may have caused the model to not prioritize that line since it doesn’trecognize the musical artists. |
| [THE PATIENT’S DAUGHTER] wasreading a umm...what’s the latest in politics with Mitch McConnell retiring.So he likes to knowabout politics. | [THE PATIENT’S DAUGHTER] was reading a what's the latest in politics with Mitch McCollum retiring. So he likes to know about politics. | {"start":START_TIME,"end":END_TIME,"text":" [THE PATIENT’S DAUGHTER] was reading a what's the latest in politics with Mitch McCollum retiring."}  [NEGLECTED MENTION OF INTEREST IN POLITICS]  {"start":START_TIME,"end":END_TIME,"text":" He likes to repair things. "} | The misspelling of Mitch McConnell resulted in exclusionas it may have affected the model’s understanding of the importance of the political figure. |
| She’s anemic, so she’s always really cold. | She’s a name, so she’s always really cold. | {"start":START_TIME,"end":END_TIME,"text":" She’s a name, so she’s always really cold "} | Transcribing “anemic” as “a name” is the major error in this example. We hypothesize theadjacent detail that the patient is cold was important enough that the line stayed in the final output, regardless of the mistake. |
