# Supplementary material for: Large Language Model–Enabled Editing of Patient Audio Interviews From “This Is My Story” Conversations: Comparative Study
Source: JMIR Med Inform. 2026 Jan 9;14:e80205. doi: 10.2196/80205 (PMC12788710; doi:10.2196/80205)
Supplement: Multimedia Appendix 3 [file medinform-v14-e80205-s003.doc]

**Figure S1**. Mean ratings for each question across all 24 interviews. Each question corresponds to the numbering indicated in Table 1 within the manuscript.

| 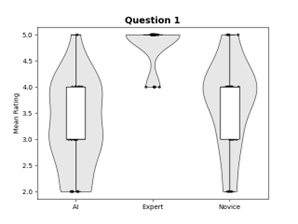 | 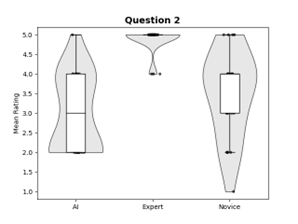 |
| --- | --- |
| 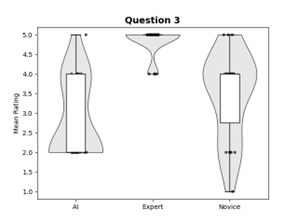 | 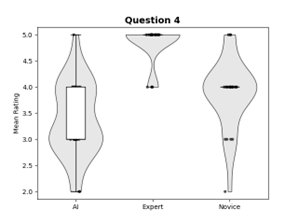 |
| 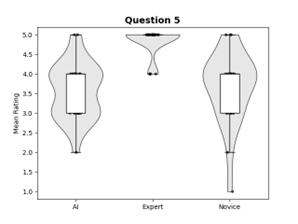 | 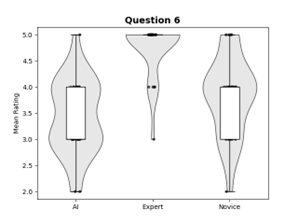 |
| 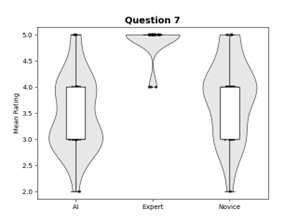 | 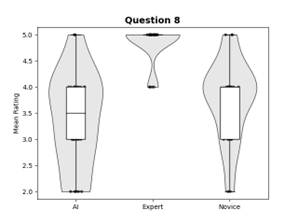 |
| 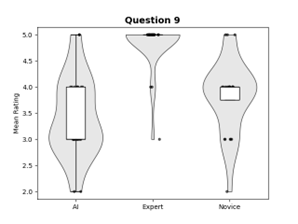 | 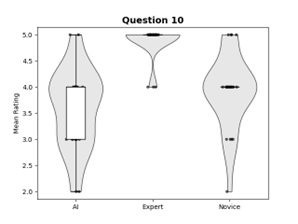 |
| 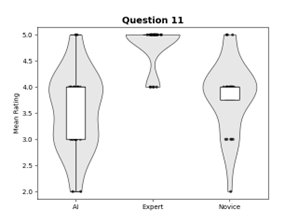 | 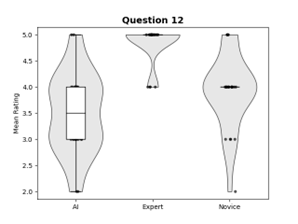 |
| 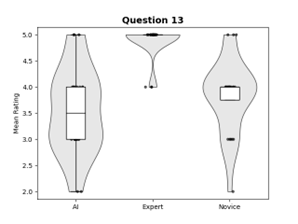 | 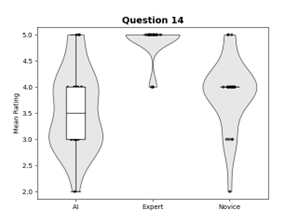 |

**eFigure 2.** Transcript length vs. all lexical and semantic similarity score regressions for AI-edited and novice-edited interviews. For all *P*-value comparisons to a slope of zero, the difference was significant (*P* < 0.050). All ANCOVA interaction *P*-values between AI and novice were not significant (*P* > 0.050).

| 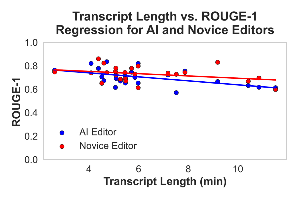 | 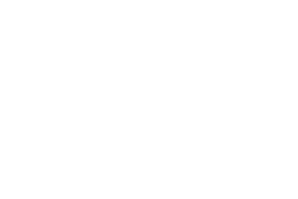 |
| --- | --- |
| 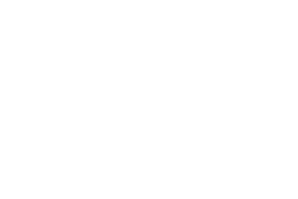 | 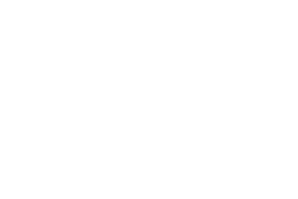 |
| 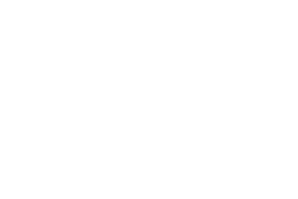 | 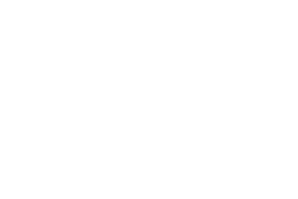 |
